# Simultaneous diagnosis of tuberculous pleurisy and malignant pleural effusion using metagenomic next-generation sequencing (mNGS)

**DOI:** 10.1186/s12967-023-04492-x

**Published:** 2023-09-30

**Authors:** Fudong Xu, Qingfeng Wang, Nana Zhang, Xuya Xing, Zichen Liu, Kun Li, Yutong Ma, Qiuxiang Ou, Yaqiong Jia, Xuejing Chen, Chen Zhang, Junhua Pan, Nanying Che

**Affiliations:** 1 Department of Pathology, Beijing Tuberculosis and Thoracic Tumor Research Institute, Beijing Chest Hospital, Capital Medical University, No. 9 Beiguan Street, Tongzhou District, Beijing, 101149 China; 2 Department of Tuberculosis, Beijing Tuberculosis and Thoracic Tumor Research Institute, Beijing Chest Hospital, Capital Medical University, Beijing, China; 3 Research & Development, Dinfectome Inc., Nanjing, Jiangsu China; 4 Department of Science and Technology, Beijing Tuberculosis and Thoracic Tumor Research Institute, Beijing Chest Hospital, Capital Medical University, No. 9 Beiguan Street, Tongzhou District, Beijing, 101149 China

**Keywords:** Tuberculous pleurisy, Pleural effusion, Malignant neoplasm, mNGS, Copy number variant

## Abstract

**Background:**

Metagenomic next-generation sequencing (mNGS) has become a powerful tool for pathogen detection, but the value of human sequencing reads generated from it is underestimated.

**Methods:**

A total of 138 patients with pleural effusion (PE) were diagnosed with tuberculous pleurisy (TBP, N = 82), malignant pleural effusion (MPE, N = 35), or non-TB infection (N = 21), whose PE samples all underwent mNGS analysis. Clinical TB tests including culture, Acid-Fast Bacillus (AFB) test, Xpert, and T-SPOT, were performed. To utilize mNGS for MPE identification, 25 non-MPE samples (20 TBP and 5 non-TB infection) were randomly selected to set human chromosome copy number baseline and generalized linear modeling was performed using copy number variant (CNV) features of the rest 113 samples (35 MPE and 78 non-MPE).

**Results:**

The performance of TB detection was compared among five methods. T-SPOT demonstrated the highest sensitivity (61% vs. culture 32%, AFB 12%, Xpert 35%, and mNGS 49%) but with the highest false-positive rate (10%) as well. In contrast, mNGS was able to detect TB-genome in nearly half (40/82) of the PE samples from TBP subgroup, with 100% specificity. To evaluate the performance of using CNV features of the human genome for MPE prediction, we performed the leave-one-out cross-validation (LOOCV) in the subcohort excluding the 25 non-MPE samples for setting copy number standards, which demonstrated 54.1% sensitivity, 80.8% specificity, 71.7% accuracy, and an AUC of 0.851.

**Conclusion:**

In summary, we exploited the value of human and non-human sequencing reads generated from mNGS, which showed promising ability in simultaneously detecting TBP and MPE.

**Supplementary Information:**

The online version contains supplementary material available at 10.1186/s12967-023-04492-x.

## Introduction

Clinically, patients with pleural effusions (PE) are commonly suspected of having malignant neoplasms or infectious diseases, e.g., tuberculous pleurisy (TBP) [1, 2]. Nowadays, diagnostic methods for tuberculous (TB) infection in clinics mainly include microbial culture, Acid-Fast Bacillus (AFB) test, Xpert MTB/RIF (Xpert) assay, and T-SPOT.TB test (T-SPOT). However, the diagnosis of TBP remains difficult as each approach has pros and cons. For instance, TB culturing requires a significantly long processing time (up to weeks) with a very high specificity [3]. AFB test is fast, but its sensitivity is only around 30% with a restricted ability to differentiate between TB and non-TB infection [4]. The Xpert assay is recommended by the World Health Organization, but its diagnostic sensitivity is not optimal enough [5]. Therefore, the development of optimized TB-detection assays is warranted. Metagenomic next-generation sequencing (mNGS) has become a powerful tool for broad pathogen detection [6], whose diagnostic value in TBP was also evaluated in multiple studies with higher sensitivity than conventional clinical approaches [4, 7, 8].

The identification of malignant PE (MPE) now mainly relies on pathological and cytologic examinations but with limited diagnostic sensitivity [9]. Genome instability considered an important genetic marker of malignant neoplasms has been studied widely based on various approaches, such as whole-genome sequencing and fluorescent in situ hybridization [10–12]. As a large number of human reads sequenced by mNGS are usually deleted without further interpretation, several studies explored the possibility of repurposing mNGS-derived human reads for copy number variant (CNV) analysis and cancer identification [13–15]. Herein, by taking advantage of both human and microbial sequencing reads, we evaluated the diagnostic performance of mNGS for simultaneously identifying TBP and MPE in this retrospective study.

## Methods

### Patients and study design

A total of 138 patients with PE who were diagnosed with TBP or other pathogen infections or MPE were enrolled in this study at Beijing Chest Hospital from June 2020 to July 2022. Patients’ demographic characteristics, clinical laboratory results, imaging data, and other medical records were retrospectively reviewed. This study was approved by the Institutional Review Board of Beijing Chest Hospital (Approval ID: 2021LSKY-58). All samples were obtained with the patient’s consent.

### Routine TB detection

Microbial culture using MGIT 960 system (Becton Dickinson, Sparks, MD, USA), AFB with Ziehl–Neelsen stain (BASO, Zhuhai, China), Xpert on GeneXpert system (Cepheid, Sunnyvale, CA, USA), and T-SPOT assay (Oxford Immunotec Ltd., Abingdon, UK) were routinely performed by the Department of Pathology for TB detection with PE, sputum, and/or bronchoalveolar lavage fluid (BALF) samples, according to the standard procedures and manufacturer’s protocols. Patients in the TBP-positive subgroup were: (1) showing positive TB culturing or Xpert result (defined as the test-defined TBP subgroup), which represent the gold standard of TB diagnosis according to the WHO guidelines [16, 17]; or (2) based on the comprehensive evaluation of clinical manifestations, auxiliary test results (including AFB, T-SPOT, and mNGS), and outcome assessment after TB drug administration (defined as the comprehensive diagnosis TBP subgroup).

### Malignant tumor identification

The diagnosis of MPE was confirmed by pathological examinations with either tissue biopsies or PE sediment specimens using hematoxylin and eosin stain for histomorphology.

### Non-TB infection

Non-TB infection patients had either positive laboratory culturing or mNGS testing result for non-TB pathogen detection, or the comprehensive evaluation result based on clinical manifestations and outcome assessment after non-TB drug administration.

### mNGS for TB detection

**Fig. 1:**
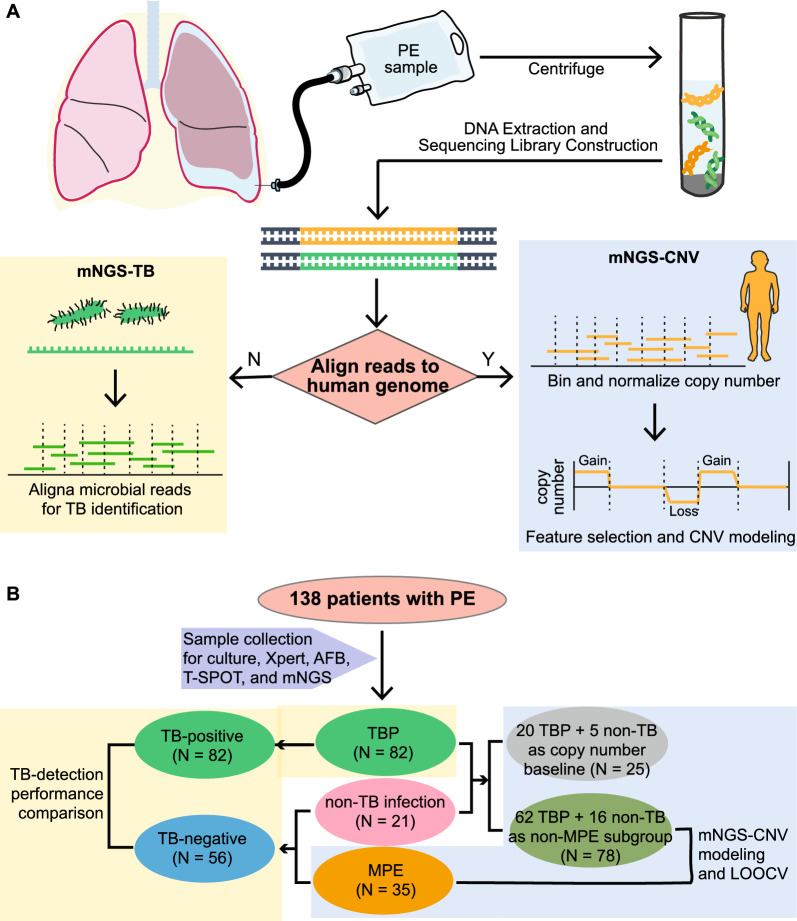
Workflow of mNGS for TB detection and malignant prediction on PE samples and the cohort overview. **A** The illustration of mNGS analysis from PE sample collection to the bioinformatic pipeline is shown, where microbial sequencing reads and human genome reads are used for TB detection (left) and CNV analysis (right), respectively. **B** The cohort overview shows the subgrouping for TB-detection performance comparison (left) and mNGS-CNV modeling (right). PE: pleural effusion; TB: tuberculosis; CNV: copy number variant; TBP: tuberculous pleurisy; MPE: malignant pleural effusion; LOOCV: leave-one-out cross validation

PE samples were used for DNA extraction using the QIAamp DNeasy Blood & Tissue Kit (Qiagen). DNA libraries were constructed using the KAPA Hyper Prep kit (KAPA Biosystems) according to the manufacturer’s protocols and sequenced on Illumina NavoSeq (Illumina). The basic procedure of mNGS was illustrated in Fig. 1A.

The bioinformatic process for pathogen detection of this mNGS pipeline was described in previous studies [18, 19]. In brief, quality control for sequencing reads was conducted by removing low-quality reads, adapter sequences, and duplicated or short (< 36 bp) reads. The remaining qualified reads were first mapped to the human reference genome (hs37d5) using bowtie2 software and then the non-human reads were aligned to the microorganism genome database for pathogens identification. A sample with at least three non-overlapping reads mapped to the TB genome and over tenfold of TB reads to the no-template control was identified as TB-positive.

### mNGS-derived CNV for identifying malignant PE

Sequencing reads that mapped to the human genome were used for genome copy number analysis using the software WisecondorX [20]. We randomly selected 25 non-malignant PE samples (20TB and 5 non-TB) that served as human genome copy number baseline to identify CNV features in the remaining 113 PE samples. CNV feature filtering excluded the features that were only presented in less than 20% of samples and the remaining 2662 CNV features were included for malignant prediction using generalized linear modeling (GLM, h2o.glm function in R). Model performance was evaluated by the leave-one-out cross-validation (LOOCV, pROC package in R).

## Results

### Patients' characteristics

From June 2020 to July 2022, a total of 138 patients were enrolled in this study, 82 of whom were diagnosed with TBP, 21 with non-TB infection, and 35 having MPE. The clinical characteristics of patients were summarized in Table 1 and the detailed clinical and diagnostic information of each patient including final diagnosis and test results were provided in Additional file 1: Table S1. The median age for the entire cohort was 58 years old, ranging from 19 to 92, and over two-thirds (95/138) were male. Underlying diseases such as diabetes, hypertension, liver diseases, etc., were reported in approximately 60% (84/138) of patients. Blood tests for white blood cell count, plateletcrit (PCT), and C-reactive protein (CRP) levels were routinely performed.

**Table 1:** Clinical characteristics of patients enrolled in this study

Characteristics N (%)		All (N = 138)	TBP (N = 82)	MPE (N = 35)	non-TB infection (N = 21)
Age	Median [range], y	58 [19–92]	48.5 [19–92]	65 [28–85]	62 [25–85]
Sex	Female	43 (31.2)	21 (25.6)	14 (40.0)	8 (38.1)
	Male	95 (68.8)	61 (74.4)	21 (60.0)	13 (61.9)
WBC	Low	3 (2.2)	3 (3.7)	0 (0)	0 (0)
	Normal	123 (89.1)	72 (87.87)	32 (91.4)	19 (90.5)
	High	8 (5.8)	4 (4.9)	2 (5.7)	2 (9.5)
	Unknown	4 (2.9)	3 (3.7)	1 (2.9)	0 (0)
PCT	Low	6 (4.3)	4 (4.9)	0 (0)	2 (9.5)
	Normal	105 (76.1)	61 (74.4)	31 (88.6)	13 (61.9)
	High	23 (16.7)	14 (17.1)	3 (8.6)	6 (28.6)
	Unknown	4 (2.9)	3 (3.7)	1 (2.9)	0 (0)
CRP	Normal	26 (18.8)	10 (12.2)	11 (31.4)	5 (23.8)
	High	109 (79.0)	70 (85.4)	23 (65.7)	16 (76.2)
	Unknown	3 (2.2)	2 (2.4)	1 (2.9)	0 (0)
Underlying diseases*	Not reported	54 (39.1)	38 (46.3)	12 (34.3)	4 (19.0)
	Diabetes	20 (14.5)	9 (11.0)	7 (20.0)	4 (19.0)
	Hypertension	33 (23.9)	9 (11.0)	15 (42.9)	9 (42.9)
	Cardiovascular disease	6 (4.3)	2 (2.4)	1 (2.9)	3 (14.3)
	Liver disease	21 (15.2)	15 (18.3)	2 (5.7)	4 (19.0)
	Renal disease	5 (3.6)	4 (4.9)	1 (2.9)	0 (0)
	Hyperuricemia	20 (14.5)	16 (19.5)	1 (2.9)	3 (14.3)
	Hyperlipidemia	9 (6.5)	5 (6.1)	4 (11.4)	0 (0)
	Respiratory failure	4 (2.9)	3 (3.7)	1 (2.9)	0 (0)
	COPD	2 (1.4)	1 (1.2)	0 (0)	1 (4.8)
	Hyperbilirubinemia	2 (1.4)	1 (1.2)	0 (0)	1 (4.8)
	Hypercholesterolemia	1 (0.7)	0 (0)	0 (0)	1 (4.8)

WBC: white blood cell; PCT: plateletcrit; CRP: C-reactive protein; COPD: chronic obstructive pulmonary disease; TBP: tuberculous pleurisy; MPE: malignant pleural effusion

*34 patients (17 TBP, 8 MPE, and 9 non-TB infections) had more than one underlying diseases

### TB-detection performance comparison between mNGS and clinical tests

**Fig. 2:**
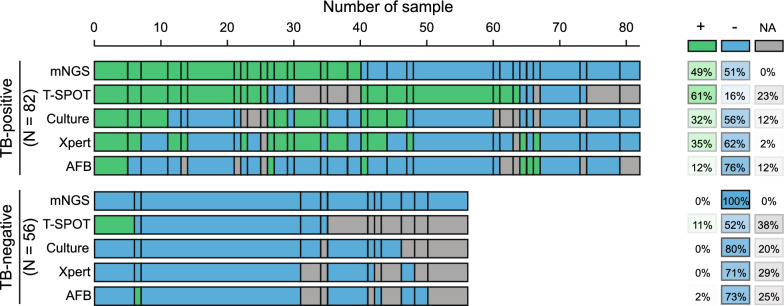
TB-detection results of mNGS and conventional clinical tests. Positive and negative detection of TB of each method is labeled in green and blue, respectively. The top panel represents the clinically diagnosed TBP patients and the bottom includes 35 MPE and 21 non-TB infected patients as the TB-negative subgroup. Positive and negative detection rates are shown on the right with scaling colors

In this study, multiple clinical tests including culture, AFB, Xpert, and T-SPOT, as well as mNGS using PE samples were performed for TB detection. Due to the nature of the retrospective clinical study, the results of culture, AFB, T-SPOT, and Xpert were undetermined in 21, 24, 40, and 18 patients, respectively (Additional file 1: Table S1; Table 2). In the TB-positive subgroup (N = 82), over 45% of patients (37/82) were defined as test-defined TBP, who had either positive TB culturing or Xpert-positive. While the remaining 45 TBP patients (55%) were diagnosed based on comprehensive clinical evidence (see Methods). In comparison, the T-SPOT assay demonstrated the highest positive detection rate (61%) among all clinical tests (culture 32%, Xpert 35%, and AFB 12%), which was also slightly higher than that of mNGS (49%, Fig. 2). Notably, no false positive TB-detection events were observed in the TB-negative subgroup (N = 56) using mNGS, culture, and Xpert assays. But the false positive rate of the T-SPOT assay reached up to 11%, which was well above other approaches (AFB: 2%).

**Table 2:** TB-detection performance comparison between mNGS and clinical tests

	TB-positive (N = 82)	TB-negative (N = 56)
mNGS		
+	40	0
−	42	56
NA	0	0
Culture		
+	26	0
−	46	45
NA	10	11
AFB		
+	10	1
−	62	41
NA	10	14
T-Spot		
+	50	6
−	13	29
NA	19	21
Xpert		
+	29	0
−	51	40
NA	2	16

Among the 52TB-positive patients whose test results were available for all five TB-detection methods, only five of them (9.6%) showed consistently positive results on all tests. Approximately 63.5% (33/52) of them had at least two positive results from the five methods (Fig. 2).

### mNGS CNV modeling for identifying malignant PE

**Fig. 3:**
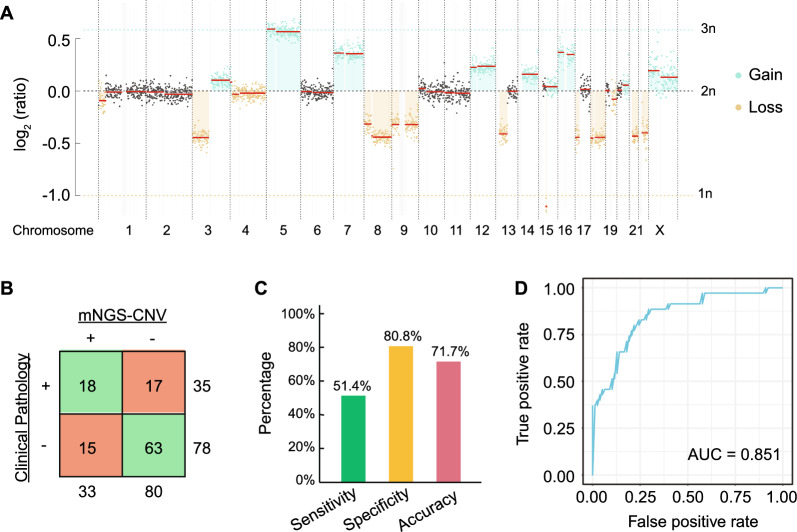
Diagnostic performance of mNGS-CNV analysis. **A** A representative chromosome copy number plot of an MPE patient with both copy number gain and loss events. **B** A contingency table shows the mNGS-CNV modeling results compared to clinical pathological diagnosis. **C** The sensitivity, specificity, and accuracy of mNGS-CNV prediction based on **B** are shown by the bar plot. **D** The Receiver Operating Characteristic (ROC) curve shows the performance of mNGS-CNV LOOCV result with an area under the curve (AUC) of 0.851

To take advantage of the human genome sequencing reads obtained from mNGS, we developed an mNGS-CNV pipeline to assess the genome copy number along the chromosomes. As described in the Methods section, 25 non-MPE samples were randomly chosen as the baseline to normalize chromosome copy number in the remaining 113 patients (35 MPE and 78 non-MPE). As shown in Fig. 3A, CNV events (both copy number gain and loss) were frequently observed in the representative patient with MPE.

GLM was performed to construct a prediction model using the filtered CNV features (frequency ≥ 20%), the predictive power of which was evaluated by LOOCV. Compared to the clinical pathology diagnosis, the mNGS-CNV modeling demonstrated 51.4% sensitivity, 80.8% specificity, and 71.7% accuracy (Fig. 3B, C), with an area under the curve (AUC) of 0.581 based on the receiver-operating characteristic (ROC) curve (Fig. 3D).

## Discussion

In this retrospective study, we explored the diagnostic utility of mNGS in detecting TBP and MPE simultaneously using a single PE sample. In terms of TB diagnostic performance, mNGS produced a sensitivity of 49% and a specificity of 100% on PE samples, which was comparable to previous clinical studies [7, 8]. Shi et al. reported that the diagnostic performance of mNGS on BALF samples was the best (sensitivity 47.9%) compared to conventional microbiological tests (sensitivity from 29.2% to 46.8%) with BALF or sputum samples [8]. Another prospective study using various clinical samples (BALF, PE, cerebrospinal, ascites, etc.) demonstrated an overall sensitivity of 44% and a specificity of 98% of mNGS on all sample types [7]. They also mentioned that positive blood T-SPOT results were observed in 82% of patients with active TB infection and 33% of those without. The relatively high false positive rate of T-SPOT makes it unsuitable serving as a stand-alone tool for diagnosing TB infection, but could be a complementary diagnostic method [21]. In our cohort, T-SPOT produced the highest sensitivity and the lowest specificity among all tested approaches, suggesting the importance of combining multiple methods to detect TB efficiently and accurately in clinical practice. Similarly, AFB itself is not enough for TB diagnosis due to the sub-optimal performance [22, 23].

Previous studies have reported genomic instability as a molecular marker of malignant neoplasms with both copy number gain and loss [10], but analyzing CNV based on mNGS-derived human reads was less investigated. With this strategy, pathogen detection and malignancy prediction were simultaneous in a single experiment from sample collection to sequencing, significantly shortening the processing time, which was critical in severe conditions. Herein, we explored the diagnostic performance of mNGS-CNV modeling on MPE prediction, which showed 51.4% sensitivity, 80.8% specificity, and 71.7% accuracy. In contrast, Guo et al.[13] reported higher sensitivity (83.7%) and specificity (97.6%) of mNGS CNV analysis on lung biopsy tissue samples instead of PE. Another study using various body fluids such as BALF, PE, peritoneal fluid, etc., showed that the mNGS-CNV test was able to identify 68% of cancer patients who were negative for conventional tests [14]. Furthermore, mNGS was also proven to detect central nervous system malignant neoplasms using cerebrospinal fluids, whose sensitivity reached up to 75% with 100% specificity [15]. Together with our study, mNGS CNV analysis presented great potential in predicting malignant neoplasms with diverse sample types. Optimizing the bioinformatic pipeline may further improve the diagnostic performance but validation in larger cohorts is warranted.

Several limitations of this study need to be noted. First, as a retrospective study, the clinical TB detection tests were performed on multiple specimens, including PE, sputum, BALF, etc., the results of which were undetermined in a small number of patients. Due to the restricted cohort size, we could not split it into training and testing cohorts for mNGS-CNV modeling, especially after excluding the 25 non-MPE samples for setting genome copy number baseline. Thus, we performed LOOCV to evaluate the performance of the mNGS-CNV analysis. Lastly, this study was a pilot study for investigating the potential of repurposing human reads generated from the well-established pathogen-detection mNGS pipeline without any optimizations to better interpret human sequences. We believe further studies on experimental and bioinformatic improvements will increase the sensitivity and specificity with an external validation cohort.

## Conclusion

In conclusion, we presented the possibility of detecting TBP and MPE simultaneously using mNGS on PE specimens, with relatively good diagnostic performance. Our study promoted mNGS as a promising tool for pathogen detection and cancer diagnosis, but prospective clinical studies and large-cohort validation are needed in the future.

### Supplementary Information

**Additional file 1: Table S1.** Patient's clinical characteristics and diagnostic results.

## Data Availability

The data generated in this study has been uploaded to NCBI database (Accession: PRJNA944842).

## Abbreviations

AFB: Acid-Fast Bacillus
AUC: Area under the curve
BALF: Bronchoalveolar lavage fluid
CNV: Copy number variant
CRP: C-reactive protein
GLM: Generalized linear modeling
mNGS: Metagenomic next-generation sequencing
MPE: Malignant pleural effusion
LOOCV: Leave-one-out cross validation
PCT: Plateletcrit
PE: Pleural effusion
ROC: Receiver-operating characteristic
TB: Tuberculosis
TBP: Tuberculous pleurisy
